# Novel insights into the pathological development of dyslipidemia in patients with hypothyroidism

**DOI:** 10.17305/bjbms.2021.6606

**Published:** 2021-11-15

**Authors:** Xin Su, Xiang Chen, Hua Peng, Jingjin Song, Bin Wang, Xijie Wu

**Affiliations:** Department of Cardiology, The Xiamen Cardiovascular Hospital of Xiamen University, Xiamen, Fujian, China

**Keywords:** Dyslipidemia, hypothyroidism, angiopoietin-like protein, fibroblast growth factor, PCSK9

## Abstract

According to the previous reports, hypothyroidism has been shown to be strongly correlated with increased circulating concentrations of total cholesterol, low-density lipoprotein cholesterol, and triglycerides. Notably, thyroid hormones are confirmed to modulate the production, clearance, and transformation process of cholesterol within circulation of mammals. Moreover, emerging evidence suggests that the thyroid-stimulating hormone could also participate in modulating serum lipid metabolism independently of thyroid hormones, which further induces the pathological development of dyslipidemia. However, the underlying mechanism is still not fully elucidated. Recently, several research studies have demonstrated that the pathogenic progression of hypothyroidism-related dyslipidemia might be correlated with the decreased serum concentrations of thyroid hormones and the increased serum concentrations of thyroid-stimulating hormones. Thus, this indicates that hypothyroidism could induce dyslipidemia and its related cardio-metabolic disorder diseases. In addition, several newly identified modulatory biomarkers, such as proprotein convertase subtilisin/kexin type 9, angiopoietin-like protein, and fibroblast growth factors, might play an important role in the regulation of dyslipidemia induced by hypothyroidism. Furthermore, under the status of hypothyroidism, significantly dysfunctional HDL particles could also be observed. In the current review, we summarized the recent knowledge of the relationship between the developments of hypothyroidism with dyslipidemia. We also discussed the updated understanding of the mechanisms whereby hypothyroidism induces the risk and the development of dyslipidemia and cardio-metabolic diseases.

## INTRODUCTION

Hypothyroidism has been identified as a common cause or a strong risk factor for multiple diseases, such as depression, bradyarrhythmia, and cretinism. As two important subtypes of hypothyroidism, the clinical hypothyroidism is characterized by the increased serum concentrations of thyroid-stimulating hormone and the reduced serum concentrations of free peripheral thyroid hormones; whereas the subclinical hypothyroidism has been shown to be accompanied with normal free serum concentrations of peripheral thyroid hormones [1]. According to the epidemiological investigations, hypothyroidism has become one of the most pressing issues in the past few decades, posing serious risks to the future of human health and leading to a high mortality in the general population worldwide.

Among multiple concomitant symptoms of hypothyroidism, dyslipidemia, as increased serum levels of low-density lipoprotein cholesterol (LDL-C), very LDL-C (VLDL-C), and triglyceride (TG), is recently suggested to be strongly correlated with the occurrence of hypothyroidism [2]. Consistent with this notion, increasing evidence has demonstrated that hypothyroidism could promote the risk and the pathological development of dyslipidemia. As reported, patients with increased serum concentrations of total cholesterol (TC) presented a relatively higher prevalence of both clinical hypothyroidism and subclinical hypothyroidism compared to that within healthy individuals [3]. By contrast, the hypothyroidism patients with the serum concentrations of thyroid-stimulating hormone >10 mIU/L were confirmed to be correlated with a higher risk of cardiovascular diseases, suggesting that dysfunctional metabolism of thyroid hormones could be identified as the essential risk factor of lipid metabolic disorders [4]. Given that the process of increased serum LDL-C intruding into the sub-endothelium modified by macrophage is intimately involved in the cascade to atherosclerotic plaque formation which subsequently induces atherosclerosis and its related coronary diseases, recent focus is shifting to illustrating the serum lipid metabolism in patients with hypothyroidism and the underlying mechanisms of hypothyroidism-mediated dyslipidemia [5].

On the other hand, several novel identified modulatory biomarkers, such as proprotein convertase subtilisin/kexin type 9 (PCSK9), angiopoietin-like protein (ANGPTLs), and fibroblast growth factors (FGFs), might play an important role in the regulation of dyslipidemia induced by hypothyroidism. Furthermore, under the status of hypothyroidism, significantly dysfunctional HDL particles could also be observed. In the current review, we summarized the recent knowledge of the relationship between the developments of hypothyroidism with dyslipidemia. In addition, the updated understanding of the mechanisms whereby hypothyroidism induces the risk and the development of dyslipidemia and its cardio-metabolic diseases are also summarized.

## CHARACTERISTICS OF DYSLIPIDEMIA IN PATIENTS WITH HYPOTHYROIDISM

Due to technological advances, major breakthroughs have been made to demonstrate the characteristics of dyslipidemia in patients with hypothyroidism. As shown in previous studies, hypothyroidism presents diverse functions in modulating the serum lipid profiles [6]. In details, it has been shown that patients with subclinical hypothyroidism presented higher serum concentrations of TC, LDL-C, and C-reactive protein (CRP); meanwhile, these patients had relatively lower concentrations of nitric oxide (NO) and omentin-1 compared to those within the euthyroid individuals. Moreover, the serum concentrations of TC, LDL-C, and CRP reduced significantly, whereas serum concentrations of NO and omentin-1 increased after using l-thyroxine replacement, indicating a potential role of thyroid-stimulating hormone in the risk of developing atherosclerosis in patients with subclinical hypothyroidism [7]. Similar with these findings, another study revealed that serum concentrations of TC increased significantly in patients with subclinical hypothyroidism compared to those within the control individuals, which also suggested a potential physiological role of thyroid-stimulating hormone in modulating the metabolism of serum lipid profiles in patients with subclinical hypothyroidism [8].

On the other hand, several clinical trials have shown that the ratios of apolipoprotein B to apolipoprotein A1 (ApoB/ApoA1)-containing lipoprotein cholesterols, such as LDL-C/HDL-C and TG/HDL-C, were significantly higher compared with those within the euthyroid individuals [9,10]. In addition, the hypothyroidism patients are confirmed to be inclined to develop postprandial hypertriglyceridemia, presenting with the elevated serum concentrations of TG, TG-rich lipoproteins (TRLs), and remnant lipoprotein (RLP) [11]. Nevertheless, whether the development of hypothyroidism could influence the serum levels and metabolism of ApoA1-containing lipoprotein cholesterol, such as HDL-C, is still not unclarified. Thereby, it is necessary to conduct other large-scale clinical trials to further explore the metabolic alterations of serum ApoA1-containing lipoprotein cholesterol in patients with hypothyroidism.

**FIGURE 1:**
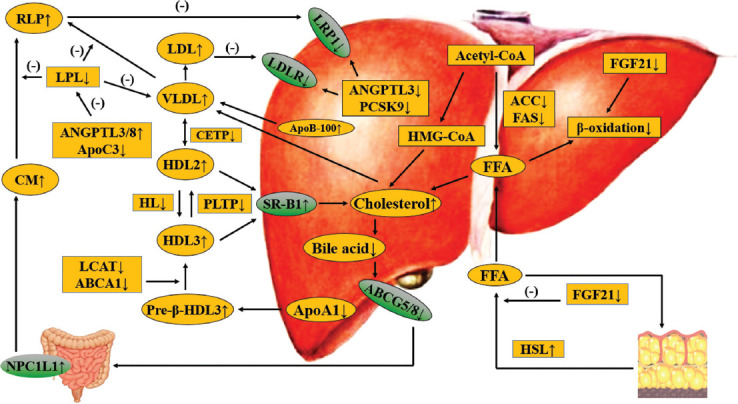
Effects of thyroid hormones and thyroid-stimulating hormones on lipid metabolism in hypothyroidism. Thyroid hormones decrease in hypothyroidism, then DNL and the activity of HMGCR reduces, leading to declined cholesterol production, but FFA β-oxidation also decreases. TH reduction reduces the activity of CYP7A1 and ABCG5/8 to reduce cholesterol clearance. In general, TG-rich VLDL level is increased in hypothyroidism, and the elevation of NPC1L1 concentration leads to an increase of TG-rich CM. DNL: de novo lipogenesis; FFA: Free fatty acid, TG: triglyceride; RLP: Remnant lipoprotein; NPC1L1: Niemann-Pick C1-like 1 protein; VLDL: Very low-density lipoprotein; ANGPTL3/8: Angiogenin-like protein3/8; ApoC3: Apolipoprotein C3; CETP: Cholesterol transport protein transporter; HL: Hepatic lipidosis; PLTP: Phospholipid transfer protein; LCAT: Lecithin cholesterol acyltransferase; ABCA1: ATP-binding cassette transporter A1; SRB1: Scavenger receptor b1; FGF19/21: Fibroblast growth factors 19/21; HMG-CoA: 3-Hydroxy-3-methyl glutaryl coenzyme A; ACC: Acetyl-CoA carboxylase; FAS: Fatty acid synthase; CM: Chylomicron; ABCG5/8: ATP-binding cassette transporter G5/8; CYP7A1: Cholesterol 7α-hydroxylase; HMGCR: HMG-COA reductase

It is also worth noting that there are significant discordances of the alterations of serum lipid profiles among the patients with clinical hypothyroidism or subclinical hypothyroidism. For instance, Dong et al. showed that in the patients with hypothyroidism, both the serum concentrations of homocysteine and LDL-C were significantly higher than those within the control individuals. With the levothyroxine treatment, the patients presented a significant decrease in the body mass index hand in hand with the serum concentrations of TC, LDL-C, and TG [12]. Another independent research demonstrated that among 11,359 participants, the thyroid function was more strongly correlated with serum lipid profiles. Mean differences in LDL-C were approximately +15.1 mg/dL and approximately +3.2 mg/dL in the patients with moderate/severe and mild chemical hypothyroidism [13]. Similar differences were seen in TG, indicating that hypothyroidism is associated with pathological development of dyslipidemia, whereas the magnitude of dyslipidemia is small in mild chemical hypothyroidism [14]. Notably, no matter whether the thyroid function is normal or not, it is certainly shown that the circulating levels of thyroid-stimulating hormone are positively correlated with circulating ApoB-containing lipoprotein cholesterol levels [15]. Hence, the higher serum concentrations of thyroid-stimulating hormones are, the greater the risks of dyslipidemia are. Taken together, we could make a reasonable speculation that the dysfunctional thyroid is strongly correlated with the progression of dyslipidemia. Besides the modulatory role of thyroid hormones, thyroid-stimulating hormone also embraces a vital function in regulating the metabolism of serum lipid profiles (Figure 1). The list of common medicines that lter the thyroid function is listed in Table 1.

**TABLE 1:**
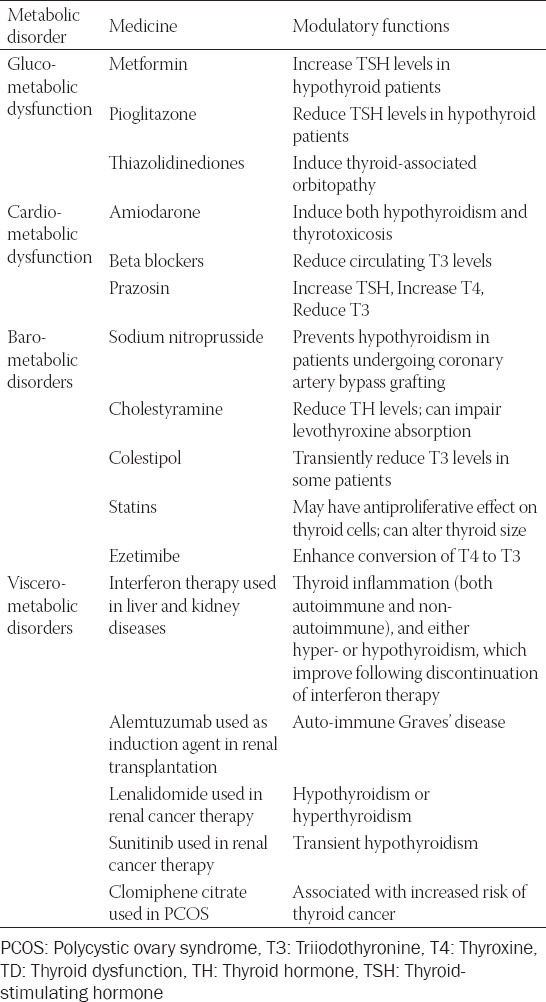
Common medicines used in cardio-metabolic disorder diseases which could alter thyroid function

## MODULATION OF LDL METABOLISM IN PATIENTS WITH HYPOTHYROIDISM

### Functions of thyroid hormones in modulating serum LDL metabolism

According to the previous study, thyroid hormones present contradictory effects on modulating the production and secretion of serum cholesterol. In detail, thyroid hormones have been shown to directly promote the expression extent of HMG-CoA reductase within hepatocytes. Since the HMG-CoA reductase is a rate-limiting enzyme in LDL-C synthesis, thyroid hormones could further facilitate the LDL-C synthesis through, at least partly, affecting HMG-CoA reductase [16]. Another independent research revealed that besides binding to thyroid hormones receptor, the triiodothyronine (T3) hormone was confirmed to activate the expression of sterol regulatory element binding protein 2 (SREBP-2) that is a major transcription factor during the adipogenesis of pre-adipocytes [17]. As excessive differentiation of adipocytes promotes the progression of obesity which is closely correlated with dyslipidemia, T3 hormone could influence the metabolism of serum LDL-C via SREBP-2. Recently, it is also shown that SREBP-2 could stimulate the gene transcription of HMG-CoA reductase, indicating another molecular mechanism whereby dysfunctional thyroid regulates the pathological development of dyslipidemia [18].

On the other hand, Sinha et al. demonstrated that thyroid hormones could stimulate the β-oxidation progress of free fatty acid and subsequently delivered the free fatty acids into mitochondria which were coupled with the promotion of hepatic autophagy, indicating that thyroid hormones could modulate serum LDL homeostasis through autophagy and upregulate oxidative metabolism [19]. Another research indicated that thyroid hormones could also stimulate the biological activity of carnitine palmitoyl-transferase I-a, which was a rate-limiting enzyme during the β-oxidation progression, and consequently modulated the metabolism of serum lipid profiles [20]. More recently, using the hypothyroidism mouse model, Zhou et al. found that compared with the pre-pregnant hypothyroidism mouse, the gestational hypothyroidism mouse presented a more prominent increase in serum LDL-C concentrations and more TG storage in hepatocytes. With in-depth investigation, the authors also found significant upregulation of SREBP-1C expressions within murine livers with gestational hypothyroidism [21]. These findings provided evidence that thyroid hormones might modulate dyslipidemia through several adipokines, such as SREBP-1C and SREBP-2. In addition, two research studies have demonstrated that thyroid hormones could also directly reduce the production of ApoB-48 and ApoB-100, which resultantly reduced the production of VLDL and chylomicron within circulation [22,23].

Aside from directly modulating the LDL metabolism, thyroid hormones have also been shown to modulate the expression levels of LDL receptor (LDLR) by binding to the thyroid-responsive element (TRE) of *LDLR* gene on the hepatocyte surface which further induced the clearance progression of circulating cholesterol and reduced the risk of dyslipidemia [24]. Likewise, SREBP-2 could combine with the sterol regulatory element (SRE) on the *LDLR* gene promoter, thereby promoting the transcription of *LDLR* gene [25]. As mentioned above, T3 hormone could activate the expression of *SREBP-2* gene, it could be speculated that thyroid hormones could modulate the *LDLR* gene expression by *SREBP-2* gene. As a result, the number of LDLR and the extent of LDL-C clearance rate decreased prominently in patients with hypothyroidism.

Besides TH, the regulatory functions of thyroid-stimulating hormone on modulating serum LDL-C concentrations have been given substantial attention in recent years. As reported, thyroid-stimulating hormone could directly affect serum LDL-C synthesis. In detail, mice with thyroid-stimulating hormone receptor deficiency exhibited lower serum TC and LDL-C concentrations compared to those within the control mice [26]. Another research indicated that the combination of thyroid-stimulating hormone and its receptor on hepatocyte membrane could facilitate the expression and biological activity of HMG-CoA reductase through the cyclic adenosine monophosphate (cAMP)/protein kinase A (PKA) signaling pathway [27]. It is also firmly reported that TSH could directly stimulate the expression of *SREBP-2* gene to regulate HMG-CoA reductase [16,28]. Consistent with this notion, in adipocytes, Moreno-Navarrete et al. found that thyroid-stimulating hormones could upregulate the expression levels of HMG-CoA reductase which subsequently influenced the intra-cellular concentrations of LDL-C [29]. Gagnon et al. demonstrated that thyroid-stimulating hormones increased the phosphorylation of perilipin and hormone sensitive lipase to promote the lipolytic progress and reduce the serum levels of LDL-C [30].

It is worth noting that thyroid-stimulating hormone has been suggested to play an important role in promoting LDL clearance. A research conducted by Song et al. showed that thyroid-stimulating hormone could induce phosphatidylinositol 3 kinase/SREBP-2 signaling pathway and further suppress the synthesis of hepatic bile acids through thyroid-stimulating hormone receptor [31]. In patients with hypothyroidism and hypercholesterolemia, serum thyroid-stimulating hormone and bile acid levels were negatively correlated which were also independent of serum thyroid hormones levels [7]. Since bile acid embraces a key role in modulating serum LDL-C levels, we could infer from these findings that thyroid hormones influence the serum LDL-C levels and the risk of dyslipidemia through affecting the synthesis and secretion of bile acid. However, it is still needed for further research to further explore the underlying mechanisms.

### Functions of thyroid hormones in modulating factors involved in hypothyroidism-induced LDL-C modulation

#### Acetyl-CoA carboxylase and fatty acid synthase (ACC/FAS)

As shown in previous studies, ACC and FAS play a catalytic role in modulating the intra-cellular metabolism of lipid profiles within the liver and the adipose tissue. Alterations of ACC/FAS could promote the risk and the development of dyslipidemia and its related cardio-metabolic disorder diseases [32]. Recently, the important roles of thyroid hormones in regulating the metabolism of ACC/FAS have begun to gain appreciation. In detail, Gambo et al. demonstrated that T3 regulated the expression of *ACC/FAS* gene through two different pathways, named the direct pathway and the indirect pathway. As reported, T3 could directly upregulate the expression levels of *ACC/FAS* gene through the TRE [33]. On the other hand, T3 has also been shown to combine with SREBP-1C/carbohydrate response element-binding protein element binding protein (ChREBP) which subsequently influenced the intracellular metabolism of ACC or FAS, indicating a unique mechanism whereby thyroid hormones influenced the serum metabolism of lipid profiles [34]. Importantly, it has also been shown that the thyroid-stimulating hormone could modulate the expression of ChREBP within hepatocytes and adipocytes [35]. As the gene expression levels of *ACC* and *FAS* could be positively regulated by ChREBP, we could make a reasonable speculation that thyroid-stimulating hormones could also modulate the metabolism of lipid profiles through affecting *ChREBP* gene expression [36]. In conclusion, these observations suggest that thyroid hormones and thyroid-stimulating hormone could significantly affect serum lipid metabolism by regulating the expression of *SREBP-1C* gene and *ChREBP* gene.

#### FGF-21

The FGF family contains 22 structurally similar proteins. According to the diverse sequence homology and phylogeny, it could be divided into seven subgroups which is classified into paracrine FGFs (including FGF-1 to FGF-10, FGF-16 to FGF-18, and FGF-20/22), endocrine FGFs (including FGF-15/19/21/23), and intracrine FGFs (including FGF-11 to FGF-14), which have been shown to be involved in a wide variety of biological metabolic processes [37]. Among different members, FGF21 is a novel discovered cytokine which is significantly increased under the status of multiple lipid disorder diseases, such as dyslipidemia, obesity, and coronary artery diseases (CADs) [38]. Recently, emerging evidence demonstrated that FGF-21 plays an important role in regulating carbohydrate, lipid and phosphate metabolism, and consequently modulating the pathological progression of lipid metabolic disorder diseases [39]. Consistently, treatment with FGF-21 has been confirmed to improve the energy metabolism in hepatocytes in both rodents and non-human primates, supporting that FGF21 could be considered as a potential therapeutic method for dyslipidemia [40].

Now that it has been firmly established that FGF-21 is associated with serum lipid metabolism, recent focus is shifting towards elucidating the relationship between FGF-21 and the alterations of serum LDL-C in patients with hypothyroidism. As reported, studies have shown that the serum FGF-21 concentrations were significantly lower in hypothyroidism patients whereas increased or did not change in patients with hyperthyroidism [41,42]. Another study has found that circulating FGF-21 concentrations were significantly increased in hypothyroidism patients which were closely correlated with the serum concentrations of thyroid-stimulating hormone, suggesting that thyroid-stimulating hormone could also regulate FGF-21 [43]. Nevertheless, the accurate relationship between thyroid-stimulating hormone and FGF-21 is needed to be further elucidated by more studies.

Increasing evidence has demonstrated that T3 could up-regulate the gene and protein concentrations of FGF-21 within hepatocytes in mice through the combination between thyroid hormone receptors with TRE in the intron 2 of *FGF-21* gene [44]. Another two research demonstrated that thyroid hormones could also induce hepatic *FGF-21* gene expression and facilitate the β-oxidation through activating AMP-activated protein kinase (AMPK) and Sirtuin 1 in a proliferator-activated receptor α (PPARα)-dependent manner in mice [45]. In mice treated with exogenous T3, the expression of FGF-21 increased significantly in a dose-dependent manner [46]. By contrast, another research has found that the peripheral administration of FGF-21 could decrease serum concentrations of thyroid hormones [47].

On the other hand, it is worth noting that FGF-21 could reduce circulating levels of free fatty acids by inhibiting lipolysis in white adipose tissue (WAT) [48] and stimulating free fatty acids uptake into WAT, which consequently suppresses the secretion of VLDL by hepatocyte [38]. Treatment with recombinant FGF-21 protein could reduce serum and hepatic TG concentrations in diet-induced obese mice by inhibiting SREBP-1C [49]. Recently, two independent researchers using mice which were treated with recombinant FGF-21 protein and found that the serum concentrations of TG, VLDL-C, and LDL-C were significantly reduced in rodents [50,51]. Gaich et al. found that in patients who were injected with FGF-21 mimetic peptide, the serum concentrations of LDL-C and TG were significantly lower whereas the serum concentrations of HDL-C were higher compared with those in the control individuals [52]. Using Ad-ChREBP-infected mice, the authors found that these mice presented decreased serum TG and VLDL-C concentrations, consistent with increased FGF-21 gene and protein levels. Infection of ChREBP could increase uncoupling proteins 1 gene contents with increased serum FGF21 levels within WAT. Given that FGF-21 could stimulate lipolysis in adipocytes, ChREBP appears to modulate serum TG concentrations by affecting FGF-21 expression levels [53]. Taken together, these findings put forward several potential mechanisms whereby FGF-21 induces dyslipidemia in patients with hypothyroidism.

FGF19, secreted from the ileum after the stimulation of bile acid, participates in the negative feedback regulation of bile acid synthesis by inhibiting hepatic cholesterol 7α-hydroxylase [54]. Thyroid hormones have been shown to play a direct role in modulating the secretion of FGF-19. Consistent with this notion, circulating FGF-19 concentrations significantly increased in patients with hypothyroidism which was independently correlated with serum concentrations of thyroid-stimulating hormone [55]. Another study showed that SREBP downregulated the expression levels of *FGF-19* gene [56]. However, whether SREBP is the underlying mechanism by which FGF-19 induces dyslipidemia in patients with hypothyroidism still needs more research.

#### MicroRNAs

MicroRNAs, containing approximately 21 nucleotides in length, are widely considered as the post-transcriptional modulators of gene expression, which are shown to affect multiple processes in eukaryotic organisms [57]. Indeed, the functions of microRNAs in modulating the pathological development of cardio-metabolic disorders, such as dyslipidemia, have been given substantial attention in the past several decades. For instance, a number of microRNAs influence HDL metabolism, from synthesis to clearance [58]. Increasing evidence also shed light on the essential role of microRNAs, including microRNA-148, microRNA-128, or microRNA-30, in modulating plasma LDL-C levels and controlling VLDL secretion [59]. For that reason, in patients with hypothyroidism, it has been proposed that microRNAs may hold functionality and potentiality in controlling the development of dyslipidemia.

The previous studies have shown that thyroid hormones could regulate cholesterol synthesis through microRNAs. For instance, Yap et al. used a microRNAs microarray to explore whether diverse microRNAs could be directly regulated by thyroid hormones in a human hepatic cell line. As demonstrated, microRNA-181 was confirmed to be modulated by thyroid hormones. With in-depth investigations, the authors also verified two novel thyroid hormone-regulated target genes which were downstream of microRNA-181, including caudal type homeo-box 2 and sterol O-acyltransferase-2. Given that sterol O-acyltransferase-2 has been identified to produce cholesteryl esters which are subsequently packaged into lipoproteins, these findings indicated that microRNA-181 embraced an essential function in modulating the metabolic genes by thyroid hormones within the hepatocyte [60]. On the other hand, Zheng et al. used the human HepG2 cells and the serum obtained from 12 Chinese hyperthyroidism patients, and found that serum microRNA-206 was significantly decreased in patients with hyperthyroidism compared with the control individuals. Treatment of HepG2 cells with T3 led to significantly decreased intracellular TG concentrations and decreased expression levels of microRNA-206. In addition, suppression of endogenous microRNA-206 expression decreased intracellular TG levels within HepG2 cells. On the contrary, over-expressing microRNA-206 within HepG2 could partially inhibit the reduction in TG concentrations resulting from treatment with T3 [61]. Conclusively, these findings demonstrated that diverse microRNAs could modulate the lipid metabolism in patients with hypothyroidism. Due to the lack of literature, whether other microRNAs could also play an important role in influencing the progression of dyslipidemia in patients with hypothyroidism is still needed to be more explored and elucidated.

#### PCSK9

At present, a clear epidemiological link between dyslipidemia and cardiovascular risk has been well-established. Among several novel-discovered candidate mediators, PCSK9 is attracting a growing attention in the past decades, due to a combined effect on lipid metabolism and inflammatory response. As demonstrated, PCSK9 is a serine protease which combines to the LDLR on the hepatocyte surface and subsequently facilitates LDLR degradation in lysosomes [62]. By this method, PCSK9 could modulate the clearance progression of LDL-C which disrupts the serum cholesterol homeostasis and induces the pathogenic development of dyslipidemia.

On the other hand, PCSK9 could also interact with low-density lipoprotein receptor related protein 1 (LRP1). As shown in previous studies, LRP1 has been postulated to participate in numerous diverse physiological and pathological processes ranging from plasma lipoprotein homeostasis, atherosclerosis, tumor evolution, and fibrinolysis to neuronal regeneration and survival [63]. Aside from the direct functions in modulating serum metabolism of lipid profiles, LRP1 could also compete with LDLR and induce the risk of dyslipidemia [64]. Consistent with this notions, emerging evidence has shown that thyroid hormones significantly downregulated serum PCSK9 concentrations [65]. With in-depth investigations, it has been shown that both SREBP-1C and SREBP-2 could affect the expression levels of PCSK9 through binding to the SRE-1 site on the *PCSK9* gene promoter [66]. Since thyroid hormones could interact with SREBP-1C and SREBP-2, we could infer from these results that thyroid hormones reduced PCSK9 concentrations though these two modulators. By this method, thyroid hormones play an important role in modulating the gene expression of *LDLR* and promoting the clearance of cholesterol from the plasma.

At present, increasing evidence has also found that the serum concentrations of thyroid-stimulating hormone is significantly positively correlated with serum PCSK9 levels, which may be partly dependent on the gene expression of *SREBP-1C* and *SREBP-2* [67,68]. It is necessary to conduct more clinical studies to further explore the relationship between thyroid-stimulating hormones with PCSK9. Besides, the mechanisms whereby thyroid-stimulating hormones modulate the serum levels of PCSK9 and the risk of dyslipidemia in patients with hypothyroidism should also be further investigated.

## MODULATION OF TG METABOLISM IN PATIENTS WITH HYPOTHYROIDISM

### Functions of thyroid hormones in modulating serum TG metabolism

As shown in previous studies, TG comes from circulating exogenous or intracellular free fatty acids which are produced by glycolysis and fat mass. The roles of thyroid hormones in modulating serum TG metabolism have been explored in recent decades. Importantly, thyroid hormones could reduce the production of VLDL-TG within hepatocytes [69]. Rodríguez et al. demonstrated that T3 played an important role in up-regulating the ApoA5 mRNA and protein concentrations in hepatocytes. Since the ApoA5 is a key determinant of serum TG concentrations which has been identified as a major risk factor for CAD and a biomarker for the metabolic syndrome, it is well-accepted that thyroid hormones influenced VLDL-TG metabolism through ApoA5, indicating that the identification of ApoA5 as a T3 target gene provides a new potential mechanism whereby thyroid hormones could influence serum TG homeostasis [70]. Martinez-Triguero et al. demonstrated in patients with hypothyroidism, the serum concentrations of TC, TG, HDL-C, LDL-C, ApoA1, and ApoB-100 decreased after thyroid hormone replacement treatment. Meanwhile, the serum Lp(a) concentrations decreased significantly after treatment of thyroid hormones. According to the results, hypothyroidism is associated with the risk of dyslipidemia, and a reduction in lipid and lipoprotein metabolism after thyroid hormone replacement which further resulted in a less atherogenic lipid profile [71]. Intriguingly, studies have also shown the impaired activity of hepatic lipidosis (HL) in hypothyroidism patients may be also related to the excessive accumulation of TRL [72]. Another research found that the transferring of TG to HDL was significantly impaired in patients with subclinical hypothyroidism, indicating that although intra-vascular metabolism of TRLs was normal, patients with subclinical hypothyroidism showed abnormalities in HDL metabolism which could be improved by levothyroxine treatment and achievement of euthyroidism [73].

RLP is a kind of small lipoprotein particle which contains apolipoprotein E (ApoE). Within human circulation, the intra-cellular concentrations of TG, phospholipid, ApoA1 and Apolipoprotein C3 (ApoC3) within TRL particles gradually reduced significantly by LPL and as a consequence, the TRL particles might transfer to RLP. Consistent with this notion, emerging evidence has demonstrated that hypothyroidism is associated with increased serum levels of RLP [74]. On the one hand, the excess production of TRL particles by hepatocyte could also induce the elevated serum levels of RLP observed in hypothyroidism patients. On the other hand, LRP1 is expressed on the hepatocyte surface which could combine with ApoE, resultantly contributing to the clearance process of RLP. As reported, thyroid hormones could increase the transcription of *LRP1* gene in the mice and in the human beings which subsequently altered the circulating lipid concentrations in hypothyroid patients [75]. By contrast, another independent research has shown that the *SREBP-1* gene and *SREBP-2* gene could also downregulate the transcription of *LRP1* gene in human vascular smooth muscle cells and macrophages through combination with SRE [76]. Thereby, these findings put forward that the dysfunctional thyroid could lead to a reduction in serum LRP1 levels hand in hand with the impaired clearance process of RLP through modulating the *SREBP* gene and protein contents.

Aside from thyroid hormone, the thyroid-stimulating hormone has also been shown to promote the synthesis of TG. In detail, Ma et al. found that the thyroid-stimulating hormone could combine with the thyroid-stimulating hormone receptors which subsequently facilitate the synthesis of TG within differentiated adipocytes through AMPK/PPARγ signaling pathway [77]. Furthermore, the thyroid-stimulating hormone could significantly increase serum TG levels within hepatocytes through TSHR/cAMP/PKA/PPARα and PPARα/AMPK/SREBP-1C signaling pathways [78]. Recently, Moon et al. showed that in euthyroid individuals, the higher levels of thyroid-stimulating hormone may affect TG metabolism through modulating the serum concentrations of ApoE, which may explain the serum ApoE levels could increase in patients with hypothyroidism [79].

### Functions of thyroid hormones in modulating factors involved in hypothyroidism-induced TG modulation

#### ANGPTLs

It is recently well-established that among the eight members of ANGPTLs family, ANGPTL3, ANGPTL4, and ANGPTL8 have been given substantial attention since they could modulate TG metabolism by affecting the biological activity of LPL. According to the reports, diverse members of ANGPTLs have similar structure and different functions. As demonstrated, these three ANGPTL proteins share a common structure whereas they differ in tissue expression [80]. Given their established role in modulating serum TG metabolism and the risk of hypertriglyceridemia, ANGPTLs have been also considered for the treatment of hypertriglyceridemia and its related atherosclerotic cardiovascular diseases. To be more specific, ANGPTL3, encoded by *ANGPTL3* gene, is produced mainly by the hepatocytes while with minor expression by the renal cells. In addition, it is also demonstrated that the ANGPTL3 protein is a 460-amino-acid peptide which includes a distinctive signal peptide sequence, an N-terminal coiled-coil domain, and a C-terminal globular fibrinogen homology domain [81]. At present, thyroid hormone has been confirmed to inhibit the expression of *ANGPTL3* gene [82]. By contrast, the circulating levels of VLDL-TG and LDL-C declined significantly in patients who carried the loss of function (LOF) mutation of *ANGPTL3* gene [83]. When the serum levels of ANGPTL3 were up-regulated, it could crack LPL though Furin protease which inhibited the catalytic activity of LPL [84]. In addition, other studies also demonstrated that ANGPTL3 could inactivate the LPL activity by catalyzing the irreversible unfolding of its hydrolase domain [85]. Using the mice with *ANGPTL3* gene deficiency, the authors of two independent researchers found that these mice exhibited declined postprandial lipid levels which were possibly due to the accelerated catabolic metabolism of TRLs and the reduced flow of free fatty acids into the hepatocyte [86]. Similar results could also be observed from the research which used the treatment of ANGPTL3 mono-clonal antibody. However, it is proposed that the transformation from VLDL to LDL is reduced partially since the enhanced clearance progression of ApoB [87]. In 2019, Yang et al. demonstrated that the serum levels of ANGPTL3 were significantly increased in the hypothyroid patients compared to those within the control individuals. However, there were no significant differences in serum levels of ANGPTL4. In addition, the positive correlations were identified between ANGPTL3 and HDL-C, and there was a negative correlation between ANGPTL3 and T3 levels, indicating that serum levels of ANGPTL3 are increased in patients with clinical and subclinical hypothyroid. Furthermore, it has been proposed that ANGPTL3 could also serve as possible biomarkers of hypothyroid disease [88].

ANGPTL8, encoded by *Gm6484* gene in mice and *C19* gene in humans, is produced by the liver and the adipose tissue. According to the reports, the circulating levels of ANGPTL8 were significantly increased under the status of hypothyroidism, which has also been confirmed to be positively correlated with serum concentrations of TG and TC [89,90]. In patients with Graves’ disease, the serum levels of ANGPTL3 were positively correlated with the serum concentrations of thyroid-stimulating hormone [91]. Another research showed that within HepG2 cells, the expression extent of *ANGPTL8* gene could be induced by thyroid hormones [92]; meanwhile, its expression could also be specifically activated through SREBP-1C and SREBP-2 in the mice liver [93]. By analyzing the mice with *ANGPTL8* gene deficiency, the clearance rate of TG increased significantly due to the facilitated biological activity of LPL, concurrently reducing the serum concentrations of TG [94]. On the other hand, using the mouse model, ANGPTL8 could interact with ANGPTL3 and promote the combination between ANGPTL3 with LPL. By this method, ANGPTL8 could promote lysis progression of LPL, leading to the increased serum TG concentrations [95]. However, due to the lack of literature, the accurate mechanism whereby ANGPTL8 modulates the risk and the development of dyslipidemia are needed for more investigations.

ANGPTL6 is recently being paid attention as it also plays an important role in modulating the risk of dyslipidemia in patients with hypothyroidism [96]. Similar to this finding, another research also confirmed that the serum ANGPTL6 concentrations were increased significantly and were positively correlated with the serum levels of thyroid-stimulating hormone and LDL-C under the pathological status of hypothyroidism [97]. Moreover, it has been shown that the serum ANGPTL6 concentrations were identified as an independent predictor of reduced serum HDL-C contents and increased serum TG contents, which could be considered as the characteristic of hypertriglyceridemia [98]. With in-depth investigation, it was found that ANGPTL6 could promote the gene expression of PPARα through the extracellular regulation of protein kinases/mitogen-activated protein kinase signaling pathway, which further lead to the enhanced gene expression of *FGF21* and resultantly promoted the β-oxidation process [99]. Given that serum FGF21 levels are more prone to be decreased under the status of hypothyroidism as described above, it could be proposed that that the thyroid-stimulating hormone could induce *FGF21* gene expression though ANGPTL6 whereas the thyroid hormones might have opposite effect on modulating serum concentrations of FGF21 compared with thyroid-stimulating hormone.

Since the gene domains are similar with those within *ANGPTL3* gene cluster, *ANGPTL4* gene has also been demonstrated to inhibit the biological activity of LPL and facilitate the disruption of serum TG metabolism [100]. On the contrary, another study also showed that the average serum concentrations of ANGPTL4 were extremely low which could not influence the LPL activity [101]. Notably, the functions of ANGPTL4 in modulating dyslipidemia were discordant according to the results provided by diverse results. For instance, two clinical trials found that the LOF mutation of *ANGPTL4* gene was closely associated with decreased serum TG levels and increased HDL-C levels [102], whereas another study demonstrated ANGPTL4 could not significantly modulated the serum TG concentrations in patients with hypothyroidism [88]. Concerning the discordant results, we still need to conduct more large-scale clinical trials and in-depth basic experiments to further elucidate the essential role of ANGPTL4 in modulating the risk and the development of dyslipidemia in patients with hypothyroidism.

#### ApoC3

ApoC3, encoded by the human *APOA1/C3/A4/A5* gene cluster, has been identified as a critical regulator of serum or intra-cellular TG metabolism. As shown in previous studies, deficiency of *APOC3* gene could induce significantly decreased serum TG concentrations. Increasing evidence indicated that ApoC3 also played a modulatory role in serum remnant cholesterol and HDL-C. Moreover, large scale population genetic studies indicated that LOF mutation in *APOC3* gene conferred decreased risk of atherosclerosis and its related CAD.

It is also worth noting that the serum concentrations of ApoC3 were found to be significantly decreased in hypothyroidism mice with or without pregnancy. In details, compared with normal control mice, the mice with gestational hypothyroidism exhibited more prominent increase compared to the pre-pregnant mice with hypothyroidism in serum concentrations of LDL-C and hepatic TG accumulation [21]. Similar with these findings, another independent research showed that the suppressed expression of *APOC3* gene could result in significantly increased biological activity of LPL which subsequently caused the decreased serum TG concentrations [103]. Taken together, the net effect of ApoC3 in modulating serum lipid metabolism in patients with hypothyroidism is more inclined to inhibiting the biological activity of LPL and affecting the serum TG metabolism.

## MODULATION OF HDL-C METABOLISM IN PATIENTS WITH HYPOTHYROIDISM

### Functions of thyroid hormones in modulating serum HDL metabolism

Since the firmly established modulatory role of thyroid hormones in serum LDL-C and TG, recent attention is also paid to elucidate the functions of thyroid hormones in modulating serum HDL metabolism. According to the reports, it has been shown that synthesis progression of HDL particles decreases in the patients with hypothyroidism. In details, Dullaart et al. demonstrated a positive relationship between free Thyroxine with the formation of pre-β-HDL particles in patients with type 2 diabetes mellitus, suggesting that variations in thyroid function within the euthyroid range may influence the metabolism of pre-β-HDL particles. On the other hand, another research revealed that the thyroid hormones could strongly induced gene and protein expression levels of ApoA1; meanwhile, the thyroid hormones has been also confirmed to enhance the ability of serum to accept cellular cholesterol through the ATP-binding cassette transporter A1, which resultantly promotes the progression of cholesterol efflux from peripheral tissues to HDL by a classical pathway which is named as reverse cholesterol transport (RCT) [104]. In conclusion, this effect is most likely attributable to increases in small HDL and poor lipid-containing ApoA1 in response to thyroid hormones.

It is worth noting that homocysteine could reduce the circulating concentrations of HDL-C by suppressing the ApoA1 protein synthesis, which consequently inhibits the progression of RCT [105]. Since it has been confirmed that in mice with hypothyroidism, the serum concentrations of homocysteine were significantly increased, suggesting that the potential modulatory role of thyroid hormones in modulating serum HDL-C might be through influencing the serum levels of homocysteine. However, after thyroidectomy, the serum concentrations of ApoA1 were significantly increased in hypothyroidism patients [106]. The potential causes of contradictory results are inclined to be due to the diverse species in different research.

Interestingly, aberrant serum levels of thyroid hormones have also been shown to exhibit diverse functions on clearance process of HDL particles from circulation. Due to the advanced research, it is demonstrated that this process is significantly inhibited in patients with hypothyroidism. Further research showed that thyroid hormones would stimulate the biological activity of HL, which consequently facilitated the degradation of HDL particles and changed components of HDL particles [107]. On the other hand, serum concentrations of cholesteryl ester transfer protein (CETP) transporter have been shown to be decreased significantly in patients with hypothyroidism. Since CETP plays an important role in suppressing serum levels of HDL-C, the positive modulatory role of thyroid hormones in CETP could make a reasonable explanation which elucidates the alterations of serum HDL-C in patients with hypothyroidism [108]. In addition, suppressed biological activity of CETP and phospholipid transfer protein has been shown to induce the decreased serum contents of HDL-2 but the increased serum contents of HDL-3 in patients with hypothyroidism [109].

The role of thyroid hormones in influencing the process of RCT has also been given substantial attention in recent several decades. As confirmed in the previous research, thyroid hormones could increase the gene transcription of cholesterol 7α-hydroxylase [110]. By this pathway, thyroid hormones could promote the transformation of cholesterol into bile acid within circulation which resultantly inhibits the progression of dyslipidemia. Recently, another research found that thyroid hormones could also stimulate the secretion of bile acid from the liver and the intestine by facilitating the gene transcription of ATP-binding cassette transporter G5/8 (ABCG5/ABCG8) in rats [104]. Since this transcriptional process is the last step of RCT, we could speculate from these findings that the thyroid hormones may modulate the serum metabolism of lipid profiles through, at least partly, affecting the expression of *ABCG5/ABCG8* gene [111]. Interestingly, a report demonstrated the degradation process of HDL particles was predominantly increased under the condition of hypothyroidism. However, it is also revealed that the clearance process of cholesterol was also accelerated since the thyroid hormones could promote the gene expression of scavenger receptor b1, as another important receptor on the surface of HDL particles which embraced a vital role in RCT [112]. In a word, the discordant effects mentioned above might cancel each other out. As a consequence, the serum HDL-C levels in patients with hypothyroidism are not consistent in different clinical observation trials.

More recently, it is also well-confirmed that the thyroid-stimulating hormone is strongly associated with the activity of CETP within circulation. Notably, Triolo et al. demonstrated that the increased serum levels of CETP could significantly accelerate the lipid exchange progression between HDL particles and LDL particles, resultantly induced the dysfunctional HDL particles and the increased cholesterol ester-rich LDL particles and VLDL particles [113].

### Functions of thyroid hormones in modulating cholesterol efflux capacity of HDL particles

Aside from the serum quantity of HDL particles and the serum concentrations of HDL-C could be modulated by dysfunctional thyroid, the capacity of cholesterol efflux, as an important metric of HDL function, has shown to be impaired in patients with hypothyroidism. For instance, van der Boom et al. enrolled 17 patients who had undergone a total thyroidectomy for differentiated thyroid carcinoma and explored the HDL particle characteristics, such as nuclear magnetic resonance spectrometry), the capacity of cholesterol efflux, and the anti-oxidative capacity. As shown in the results, the patients presented increased serum concentrations of TC, HDL-C, and ApoA1. Although the contents of HDL particles were unchanged, there was a shift in HDL subtypes toward larger HDL particles. In addition, the capacity of cholesterol efflux was significantly decreased. The anti-oxidative capacity of HDL particles presented not significantly alterations [106]. The other independent research conducted by Jung et al. put forward the similar results. However, the latter research found that the activity of paraoxonase-1 (PON-1), as an important anti-oxidative enzyme, remains unaltered after thyroidectomy; the activity was significantly decreased after expressed as the PON-1/ApoA1 ratio [79]. Taken together, the above findings shed light on that in patients with hypothyroidism; dysfunctional thyroid may affect the HDL function. Nevertheless, the potential underlying mechanisms are still needed for further investigations.

## CONCLUSION

In this review, we put forward a summary of several research which has demonstrated that the characteristics of hypothyroidism-related dyslipidemia; moreover, it is also summarized that the hypothyroidism-related dyslipidemia is closely associated with the altered serum concentrations of thyroid hormones and thyroid-stimulating hormone, indicating that hypothyroidism could induce dyslipidemia and its related cardio-metabolic disorder diseases. In addition, we also listed several novel identified modulatory biomarkers which play an important role in the regulation of dyslipidemia induced by hypothyroidism, including PCSK9, ANGPTL3, ANGPTL8, FGF-21, FGF-19, and several microRNAs. Due to the eye-catching results, it is well-accepted that besides the modulatory role of thyroid hormones, the thyroid-stimulating hormone is also shown to be another important regulator in affecting the serum or intra-cellular lipid metabolism under the status of hypothyroidism. As reported, even in patients with normal thyroid and subclinical hypothyroidism, higher thyroid-stimulating hormone concentrations could also significantly affect lipid metabolism. Moreover, it seems that maintaining the serum contents of thyroid-stimulating hormone at a low normal level to minimize cholesterol concentrations could be identified as an important therapeutic strategy to hypothyroidism in daily clinical practice. Nevertheless, the main alteration of the hormones in patients with hypothyroidism is thyroid hormones, and thyroid-stimulating hormone could be influenced through a negative feedback regulation of thyroid hormones. Thereby, whether thyroid-stimulating hormone metabolic pathway makes up a large proportion in patients with hypothyroidism is still needed to be further illustrated. Our recently findings also confirmed that several regulatory factors, such as SREBPs, ChREBP, ANGPTLs, microRNAs, and FGFs, are also involved in the tissue related-lipid metabolism within the liver and the adipose tissue isolated from the patients with hypothyroidism. In addition, we also confirmed that the condition of hypothyroidism could influence HDL function, especially the modulatory role of RCT, in Chinese patients. As the results showed, these important modulatory factors could be identified as novel and potential therapeutic targets of hypothyroidism. Further basic research and clinical trials are still required to clarify the accurate functions and the respective mechanism whereby these modulatory factors influence serum or intracellular lipid metabolism which subsequently promotes the risk of dyslipidemia development in patients with hypothyroidism.
